# Comparing neonatal respiratory morbidity in neonates delivered after 34 weeks of gestation with and without antenatal corticosteroid

**DOI:** 10.12669/pjms.336.14031

**Published:** 2017

**Authors:** Burcu Kisa Karakaya, Yasemin Tasci, Ozlem Yoruk, Hatice Kansu-Celik, Fuat Emre Canpolat

**Affiliations:** 1 Burcu Kisa Karakaya, Department of Obstetrics and Gynecology, Zekai Tahir Burak Women’s Health Care Training and Research Hospital, Ankara, Turkey; 2 Yasemin Tasci, Department of Obstetrics and Gynecology, Zekai Tahir Burak Women’s Health Care Training and Research Hospital, Ankara, Turkey; 3 Ozlem Yoruk, Department of Obstetrics and Gynecology, Zekai Tahir Burak Women’s Health Care Training and Research Hospital, Ankara, Turkey; 4 Hatice Kansu-Celik, Department of Obstetrics and Gynecology, Zekai Tahir Burak Women’s Health Care Training and Research Hospital, Ankara, Turkey; 5 Fuat Emre Canpolat, Department of Neonatology Unit, Zekai Tahir Burak Women’s Health Care Training and Research Hospital, Ankara, Turkey

**Keywords:** Neonatal respiratory morbidity, Betamethasone administration, Late preterm

## Abstract

**Objective:**

To investigate the effect of antenatal corticosteroid prophylaxis on neonatal respiratory morbidity between 34 and 37 weeks of gestation.

**Methods:**

This retrospective study evaluated the neonatal respiratory complications of 683 low risk singleton pregnancies delivered at 34-37 weeks of gestation in a tertiary care center between Jan 2012 and Sept 2015. Group-I (n=294) comprised data of woman who did not receive betamethasone and Group-II(n=396) comprised those who received betamethasone after 34 weeks of gestation for cases at risk of preterm birth. Primary outcome was neonatal respiratory morbidity (NRM). NRM was defined as any respiratory disease that required medical support including supplemental oxygen, nasal continuous positive airway pressure, endotracheal intubation, or exogenous surfactant, with more than 25% oxygen for > 10 minute to maintain neonate oxygen saturation >90% Demographic characteristics, mode of delivery, fetal birth weight and neonatal respiratory complications was compared between the two groups.

**Results:**

There was no statistically significant difference for neonatal respiratory morbidity development rate between patients who received betamethasone or those who did not receive it. The incidence of neonatal respiratory morbidity was similar (15.3% in the control group and 14.9% in the intervention group; p=0.88).

**Conclusion:**

We found no improvement with betamethasone administration empirically in late preterm birth as regards prevention of Neonatal Respiratory Morbidity(NRM).

## INTRODUCTION

In current obstetric practice, the use of antenatal corticosteroids in women delivered after 34 weeks of gestation for an obstetric indication still remains controversial. Antenatal corticosteroid administration is one of the most effective interventions in perinatal medicine to increase the fetal lung maturation. Antenatal steroids exert their effect via sodium ion channels on the pulmonary epithelium through altered ion secretion. The immature lung secretes and creates a wet surface of the airways, whereas the mature lung under the steroid effect absorbs the secretions and creates an effective surface of the airway capable of gas exchange (6^th^ item). Although fetal lung development is complete, serious respiratory problems can be seen even after 34 weeks of gestation.^1^ There is no significant difference in the rate of RDS, intraventricular bleeding, neonatal death, and NICU stay between the late preterm infants receiving and not receiving antenatal steroids in the literature.

Late preterm birth is defined as the birth between the 34^th^ and 37^th^ weeks of pregnancy and accounts for 60% to 70% of the preterm births. The incidence of respiratory morbidity, including respiratory distress syndrome (RDS) and transient tachypnea of the newborn (TTN), with an increasing rate of neonatal intensive care unit (NICU) admissions, are higher in the late preterm infants, compared to term infants.^2^,^3^

There is no concensus about the use of antenatal steroids in the pregnant women who have preterm risk at 34-37 week of gestation. The American College of Obstetricians and Gynecologists (ACOG) has only recommended antenatal corticosteroids up to 34^th^ weeks of gestation.^4^ However, the Royal College of Obstetricians and Gynecologists (RCOG) recommends routine administration of antenatal glucocorticoids for all women at risk of preterm birth up to and including pregnancies between 34^+6^ and 38^+6^ weeks of gestation.^5^ The latter recommendation is based on the Antenatal Steroids for Term Caesarean Section (ASTECS) trial, which has reported a reduction in the overall incidence of respiratory problems in the group treated with betamethasone (composite of transient tachypnea of the newborn and respiratory distress syndrome 2.4 vs 5.1%; RR 0.46, 95% CI 0.23-0.93).^6^

Late preterm birth, compared to term delivery, has shown to be associated with an increased risk of RDS and other respiratory morbidities.^7^ The increased risk of respiratory morbidity in the late preterm infants is caused by the immature lung structure, as the lung development of the terminal respiratory sacs and alveoli continues through 34 and 36 gestational weeks, and results in an increased risk of RDS for infants of mothers who did not receive antenatal steroids, in particular.^8^ (6^th^ item)

In another study where antenatal steroids were used between 34 to 36 weeks of pregnancy, the rate of neonatal respiratory complications was found to be significantly reduced.^9^ Recent studies have shown that antenatal steroid treatment even after 34^th^ week of gestation may play a key role in preventing pulmonary complications. In this study, we aimed to investigate whether the application of betamethasone on hospitalized pregnant patients after 34 weeks of gestation is effective at reducing neonatal respiratory problems.

## METHODS

This retrospective study was conducted at the delivery unit of Zekai Tahir Burak Women’s Health Education and Research Hospital between January 2012 and September 2015. It included 683 consecutive women with a normal singleton pregnancy who delivered healthy infants between 34 and 41 weeks of gestation, 389 received betamethasone for fetal lung maturation between 34 and 37 weeks of gestation. A power analysis using the e-Picos computer program (MediCres, NY, USA) indicated that a total sample of 281 patients for each group would be needed with a 80% power with alpha at 0.05 for main determinant of neonatal respiratory morbidity proportion.(3th item). The study was approved by the Local Ethical Committee of the University of Health Sciences, Zekai Tahir Burak Women’s Health Education and Research Hospital (Institutional Board No. 26/2014, November 2014) and it was conducted in accordance with the principles of the Declaration of Helsinki.

Patients with with preexisting hypertension, pre-eclampsia, diabetes mellitus, glucose intolerance, chronic diseases, or obstetric complications such as placenta previa, intrauterine growth retardation were excluded.

The exact weeks of gestation of the patients were determined by using the last menstrual period and/or ultrasonographic measurement of the crown-rump length in early pregnancy and after 12 weeks of gestation by using ultrasonographic measurement of the fetal biparietal diameter and femur length and patients between 34^+0^and 41^+0^ weeks of gestation.

The obstetric outcomes recorded included maternal demographic characteristics, gestational age on admission, indications for admission, corticosteroids used, and mode of delivery. Neonatal outcomes included gestational age at delivery, birthweight, Apgar scores, neonatal morbidity, and duration of NICU stay. Primary outcome of this study was neonatal respiratory morbidity (NRM). NRM was defined as any respiratory disease that required medical support including supplemental oxygen, nasal continuous positive airway pressure, endotracheal intubation, or exogenous surfactant, with more than 25% oxygen for > 10 minute to maintain neonate oxygen saturation >90%. All neonates were examined by a neonatologist and the diagnosis of respiratory problems was confirmed by the Department of Neonatology. Respiratory distress was defined as the presence of signs of respiratory distress (i.e., cyanosis, grunting, inspiratory stridor, nasal flaring, or tachypnea) with retractions in the intercostal, subcostal or supracostal spaces with specific radiological features in some cases. Transient tachypnea of the newborn was also diagnosed by the attending pediatrician by the presence of tachypnea immediately or within two hours after birth, with other predictable signs of respiratory distress. For the NICU admitted cases, respiratory distress was graded according to the oximetry measurements as mild, if the infant received less than 30% oxygen, severe, if he received more than 70% oxygen or ventilator support, and otherwise moderate. The chest X-ray for the NICU-admitted cases examined the radiological features of TTN, such as diffuse parenchymal infiltrates or intra-lobar fluid accumulation and RDS, as the reticular granular pattern.

A course of therapy consists of two doses of betamethasone of 12 mg given intramuscularly 24 hours apart or four doses of betamethasone of 6 mg given intramuscularly 12 hours apart. We administered full course of therapy. Our study consist of two groups: Group-I included 294 women who did not receive betamethasone for fetal lung maturation, and Group-II included 389 women who were administered 12 mg betamethasone intramuscularly in a course of therapy (Celestone vial, Schering-Plough, Istanbul, Turkey). Patient who delivered at any time point following betamethasone administration were included and the results were recorded in minutes in each delivery.

### Statistical Analysis

Statistical analysis was performed using SPSS for Windows version 22.0 software (SPSS Inc., Chicago, IL, USA). Continuous variables were presented in mean ± standard deviation or median (min-max). Categorical variables were expressed in frequencies and percentages. The normality of the continuous variables was evaluated using the Shapiro-Wilks test. Differences between the two groups according to continuous variables were analyzed by independent samples t or Mann-Whitney U tests, if applicable. Categorical variables were compared using the Pearson chi-square or Fisher’s exact tests (4th item). A *p* value of less than 0.05 was considered statistically significant.

## RESULTS

The mean age was 30.7±5.7 years in Group-I and 27.3±5.6 years in Group-II. The mean gestational age of Group-I and Group-II was 36.3±0.9 and 36.4±1.4 weeks, respectively. The mean birthweight was 2798±291 g and 2861±430 g in Group-I and Group-II, respectively. There were no statistically significant differences in the gravidity, parity, gestational age, and birthweight between the groups (Table-I).

**Table-I:** Baseline characteristics of patients.

*Variables*	*(No bethametosone)*	*(Received betamethasone)*	*P values*
Age, years	30.7±5.7	27.3±5.6	<0.001*
Gravidity	2 (0-7)	2 (1-14)	0.418
Parity	1 (0-5)	1 (0-6)	0.775
Gestational age at delivery (w)	36.3±0.9	36.4±1.4	0.586
Birthweight, g	2798±291	2861±430	0.030*

Variables are Mean±SD.

* P<0.05, significant

A total of 132 (44.2%) neonates in Group-I and 193 neonates in Group-II were delivered by cesarean section (C/S), due to a previous scar, fetal distress, and presentation abnormalities. There was no statistical difference in the development of respiratory problems between the infants delivered vaginally and by cesarean section.

Neonatal Apgar scores were assessed in the infants of both groups. The Apgar scores were as follows: 7 and 8 at one minute and 8 and 10 at five minute in Group-I and Group-II, respectively. Group-II had higher Apgar scores, compared to Group-I, indicating a statistically significant difference (p<0.001 at one minute and p<0.001 at five minute) (Table-II).

**Table-II:** Summary of outcomes.

*Variables*	*(No bethametosone)*	*(Received betamethasone)*	*P*
***Delivery mode n (%)***			0.207
C/S	132 (44.7%)	193 (49.6%)	
Vaginal Birth	163 (55.3%)	196 (50.4%)	
***Neonatal Sex n (%)***			0.471
Female	145 (49.2%)	201 (51.9%)	
Male	150 (50.8%)	186 (48.1%)	
***Duration of delivery after betamethasone (minute)***		430 [20 – 399960]	
Apgar score 1	7 [4 – 9]	8 [5 – 9]	<0.001*
Apgar score 5	9 [0 – 10]	10 [7 – 10]	<0.001*
Respiratory Problems (RDS, TTN, Pul maladaptasyon n (%)	45 (15.3%)	58 (14.9%)	0.885
Time in intensive care unit(day)	3(1 – 20)	5(0 – 29)	0.020*

C/S, cesarean section; RDS, respiratory distress syndrome; TTN, transient tachypnea of the newborn.

* P<0.05, significant.

In addition, 45 neonates (43.7%) in Group-I and 58 (56.3%) in Group-II had respiratory problems. However, there was no statistically significant difference in the rate of respiratory problems between the two groups (Table-III).

**Table-III:** Hospitalization due to respiratory problems (RDS, TTN, pulmonary maladaption) (n=103).

*Gestational age*		*36.0±1.0*
Age		29,0±5.8
Mode of delivery n (%)	C/S	58 (56.3%)
	Vaginal delivery	45 (43.7%)
Groups	Group 1	45(43.7%)
	Group 2	58(56.3%)
Duration of delivery after betamethasone (min)		244 (20 – 23580)

C/S, cesarean section; RDS, respiratory distress syndrome; TTN, transient tachypnea of the newborn.

According to the gestational age, there was a statistically significant difference in the mode of delivery (C/S <37 gestational week; vaginal delivery >37 gestational week). In addition, according to the gestational age, there was no statistically significant relationship between betamethasone use and respiratory problems (1,774 in vaginal delivery vs 1,643 by C/S).

## DISCUSSION

In the present study, we evaluated the effect of betamethasone administration in women who were late preterm delivery (34 to 37 weeks) (2nd item) and found no significant difference in reducing risks of respiratory disorders. Our findings are similar to the triple-blind clinical trial study carried out by Fetiosa Porta et al.^10^ who reported that antenatal steroids did not reduce the risk of respiratory disorders of the late preterm infants.

The recommendations of antenatal steroid administration vary between the guidelines. The ACOG recommends antenatal steroid administration for patients until 34 weeks of gestation, whereas the RCOG recommends until 34 weeks and six days in addition to the elective C/S patients before 38 weeks and six days of gestation.^4^,^11^ In addition, the Antenatal Late Preterm Steroids (ALPS) trial of which preliminary results were announced suggested that the need for surfactant in the intensive care unit and risk of serious morbidity of respiratory disorders could be reduced with the administration of steroids in the late preterm infants.^12^

Furthermore, the period of time after antenatal steroid administration and number of dose administered to late preterm infants differ among the studies in the literature. The most common administration methods are the course of dose within 12 or 24 hours. Balci et al.^13^ administered steroids in a single dose to newborns of gestation weeks between 34 until 36 weeks, and showed a significant difference in the outcomes of respiratory disorders. In our study, we administered a course of dose of steroids. The time between steroid administration and delivery also affects the respiratory disorders-related morbidity. In the study of Sekhavat et al.^14^, if the steroid was administered in a shorter period less than two days, the need of ventilation and resuscitation of the newborns increased.

Despite the fact that antenatal steroid administration may be effective in reducing the risks related to respiratory disorders of the late preterm newborns, there are studies which show increased maternal and fetal infection rates and neonatal sepsis with worsened fetal heart rate pattern and biophysical activity. Furthermore, many late preterm pregnancies should be treated with steroids to prevent respiratory distress syndrome of a single newborn.

In a recent prospective study which includes 2827 late preterm infants, outcome shows that severe respiratory complications transient tachypnea of newborn surfactant need and bronchopulmonary dysplasia occurred significantly less in steroid administered newborns. Relatively short-term follow-up duration can be regarded as a limitation for the evaluation of the effects of antenatal steroids in this patient population. In addition to this no increase in risk of neonatal sepsis and chorioamnionitis is seen except neonatal hypoglycemia.^9^

### Limitation of the study

It was a retrospective study. Further prospective studies would have important implications for neonatal respiratory morbidity..

## CONCLUSION

This study showed no beneficial effect of betamethasone administration empiricaly in late preterm birth for preventing Neonatal Respiratory Morbidity (NRM). Further randomized controlled studies with more participants are needed to evaluate the association between betamethasone administration empirically in late preterm birth and preventing NRM.

### Authors’ Contribution

**BKK, YT, HC** conceived, designed, did statistical analysis & editing of manuscript.

**BKK, OY, HC, FEC** did data collection and manuscript writing.

**YT, FEC** did review and gave final approval of manuscript.
